# Biomechanical behavior of three types of fixation in the two-part proximal humerus fracture without medial cortical support

**DOI:** 10.1371/journal.pone.0220523

**Published:** 2019-07-30

**Authors:** Paulo Ottoni di Tullio, Vincenzo Giordano, Eder Souto, Hugo Assed, João Paulo Chequer, William Belangero, José Ricardo L. Mariolani, Hilton A. Koch

**Affiliations:** 1 Serviço de Ortopedia e Traumatologia Prof. Nova Monteiro, Hospital Municipal Miguel Couto, Rio de Janeiro, RJ, Brazil; 2 Laboratório de Biomateriais em Ortopedia, Faculdade de Ciências Médicas, Universidade de Campinas (UNICAMP), Campinas, SP, Brazil; 3 Departamento de Ortopedia, Faculdade de Ciências Médicas, Universidade de Campinas (UNICAMP), Campinas, SP, Brazil; 4 Departamento de Radiologia, Universidade Federal do Rio de Janeiro (UFRJ), Rio de Janeiro, RJ, Brazil; University of California Los Angeles, UNITED STATES

## Abstract

**Background:**

The purpose of this study was to evaluate the role of a non-locking plate applied to the anteromedial surface of the proximal humerus on loads at the implant-bone interface of non-locking and locking lateral plate fixation of proximal humeral fractures with a medial gap.

**Methods:**

Twenty synthetic humeri models were used. In fifteen, the proximal portion of the humerus was osteotomized to create a two-part surgical neck fracture, with a 10-mm medial gap and a 5-mm lateral gap; five models were controls. In the osteotomized humeri, five models were stabilized with a locking lateral plate (group L), five with a locking lateral plate and an anteromedial non-locking plate (group L+T), and five with a non-locking lateral plate and a non-locking anteromedial plate (group T+T). All humeri were tested under axial loading until catastrophic failure, which was characterized as complete closure of the medial gap. Stiffness was calculated using force vs. displacement curves. The data were analyzed via descriptive and inferential studies, at a 5% significance level.

**Results:**

Statistically significant differences were seen among all the constructions. The combination of a lateral locking plate with an anteromedial non-locking plate (group L+T) was the stiffest construction, while the combination of a non-locking lateral plate with a non-locking anteromedial plate (group T+T) was the least stiff, even in comparison with a single locking lateral plate (p = 0.01). When the two groups which utilized a lateral locking plate (groups L+T and L) were compared, the group with additional anteromedial support demonstrated greater stiffness (p = 0.03), and stiffness values for the control group comprised of intact humeri models were even higher (p = 0.01).

**Conclusion:**

Combining a lateral locking plate with a non-locking anteromedial plate provides a stiffer construction for fixation of unstable two-part proximal humerus fractures with a medial gap. Mechanical benefits of medial support with a second non-locking antero-medial plate seems to be related with better construct stability in terms of strength and fatigue, potentially reducing the risk of varus collapse of the humerus head and fracture healing disturbances.

## Introduction

Fractures of the proximal region of the humerus are common, and present numerous challenges as well as a high complication rate, despite improvements in implants and the use of less-invasive techniques [1]. Varus reduction and insufficient bone stock contribute to the high number of cases in which surgery is unsuccessful [1]. Recently several authors have called attention to the lack of medial support as an important risk factor for surgical failure [2–5]. Krappinger et al. indicated age >63 years, bone mineral density levels <95 mg/cm^3^, non-anatomic reduction, and failure to restore cortical medical support as risk factors for post-osteosynthesis failure [4].

Adequate mechanical support in the inferomedial region of the proximal humerus has been suggested as an influential factor in preventing complications related to fixation of unstable fractures with locking plates [5–9]. Jung et al. reported a significant reduction in the number of complications in a series of 62 patients (63 fractures) when medial support was restored, even when osteoporosis was present [8]. Gardner et al. proposed a fibular allograft to improve medial support and consequently minimize mechanical failures and reoperation, but they also indicated risks related to remodeling and osteointegration of the graft, as well as the greater cost of this procedure [6].

Theopold et al. evaluated the situation of large metaphyseal medial cortical defect using humerus fracture model with a two-part fracture in a biomechanical comparison of the stability of two techniques: (1) plate osteosynthesis and calcar screws and (2) hybrid double plate osteosynthesis [5]. They observed that hybrid double plate osteosynthesis tended to confer higher stiffnesses and ultimate load than the medial support screws, although this trend was below the level of significance for both variables. Despite the potential criticism of an additional plate osteosynthesis in the region of the bicipital groove, it was demonstrated that a second plate alters load distribution, fulfilling the additional benefits of a medial support screw [5]. Wanner et al. suggested the use of two non-locking plates, one on the anteromedial face and the other on the lateral face of the proximal region of the humerus, and presented a low complication rate and very positive results (88% excellent or satisfactory) [10]. However, they did not assess this arrangement in cases where there was failure of the medial column. Despite of those two studies, currently very few is known to clearly indicate the use of a second plate to augment the fixation of unstable proximal humerus fractures.

We hypothesize that adding a non-locking plate in a more anteromedial position also decreases the risk of mechanical failure and valgus subsidence in proximal humerus fractures with a medial cortical gap without damaging or invading the bicipital groove. In the current experiment the role of a non-locking plate applied to the anteromedial surface in fractures of the surgical neck of the humerus involving a medial gap fixed with a lateral plate was evaluated. The objective of this study was to compare the axial stiffness of three bone-plate constructs, two of them with a non-locking plate in an anteromedial position.

## Methods

Twenty right humeri made of synthetic bone (3012BESP, Nacional Ossos, Brazil) were used; each bone was 330 mm long, with a medullary cavity measuring 7 mm in diameter, 44 mm diameter head, 22 degrees retroversion, and cervicodiaphyseal angle of 130 degrees. The models were equally divided into four groups: three experimental groups and one control (group O), in which the specimens remained intact. In the experimental groups, a fracture was systematically created at the surgical neck of the humerus with a 10 mm medial defect and 5 mm lateral defect, according to the methodology described by He et al. [11]. A goniometer was used to mark the fracture lines and the bone was cut 35 mm from the apex of the humeral head.

In order to facilitate reduction of the fragments and subsequent positioning of the implants, the models were fixed before the fracture was created in one of the following arrangements:

1. One anatomic locking plate positioned on the lateral surface of the proximal humerus (group L, n = 5),
2. Two non-locking one-third tubular plates positioned at right angles, one on the lateral face and the other on the anteromedial face of the proximal third of the humerus (group T+T, n = 5),
3. Two plates positioned perpendicular to each other, one anatomic locking plate on the lateral face and a one-third tubular plate on the anteromedial face of the proximal third of the humerus (group L+T, n = 5).

The anatomic locking plates were T-shaped, with four holes in the upper horizontal portion and five in the lower vertical section (DPS, Johnson & Johnson Company, USA). The non-locking one-third tubular plates had four holes (Ortosintese, Brazil). The bone models were X-rayed to assess correct positioning of the plate-screw arrangement in the proximal region of the humerus. The implants were removed in order to create the fractures and then reinserted in the same arrangement. For each plate, all screws were inserted, so there was no empty hole that could reduce bone-plate stiffness (Table 1).

**Table 1:** Lengths, type (locking/non-locking), and position of screws used for each plate.

Plate	Screw length (mm)	Screw type	Screw position
T-shaped anatomic locking plate	38 40 40 40 48 28 26 26 26	L L L L L L L L L	- horizontal part, anterior hole (hole 1) - horizontal part, hole 2 - horizontal part, hole 3 - horizontal part, posterior hole (hole 4) - vertical part, superior hole (hole 1) - vertical part, hole 2 - vertical part, hole 3 - vertical part, hole 4 - vertical part, inferior hole (hole 5)
Lateral non-locking one-third tubular plate	45 45 28 26	NL NL NL NL	- vertical part, superior hole (hole 1) - vertical part, hole 2 - vertical part, hole 3 - vertical part, inferior hole (hole 4)
Anteromedial non-locking one-third tubular plate	45 45 28 26	NL NL NL NL	- vertical part, superior hole (hole 1) - vertical part, hole 2 - vertical part, hole 3 - vertical part, inferior hole (hole 4)

L–locked, NL–non-locked.

**Fig 1:**
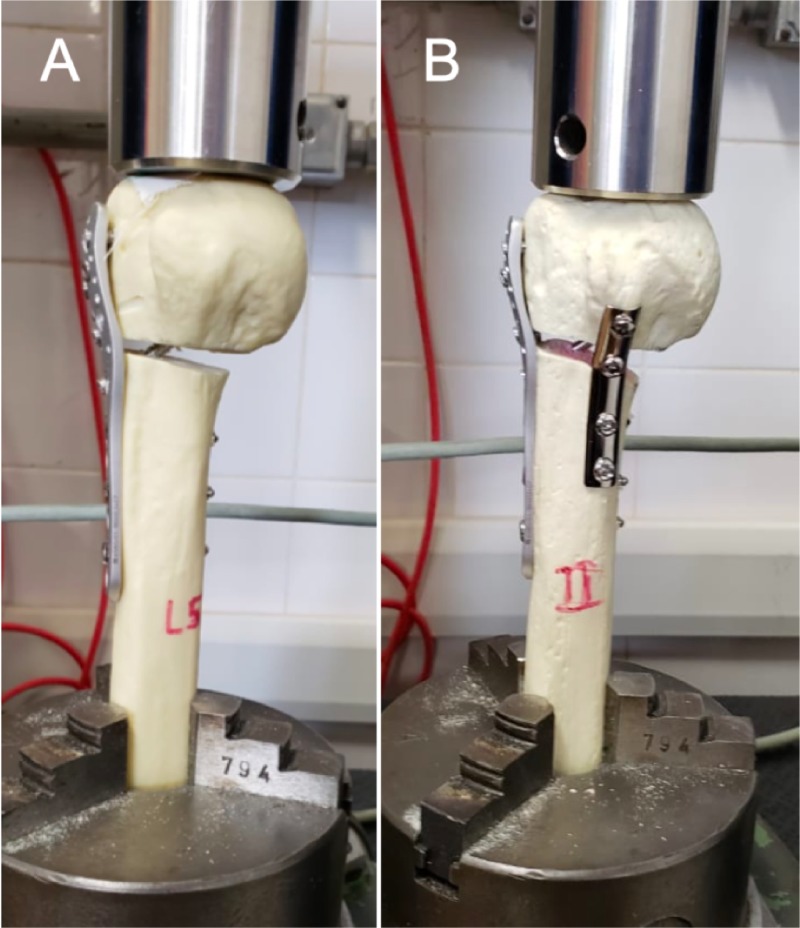
Mechanical testing. The distal portion of the bone models were cut in order to fit in the testing machine and positioned lengthwise to be tested in axial load for displacement rate. A–specimen of Group L, B–specimen of Group L+T.

The distal portion of the bone models were cut into 225 mm long sections measured from the humerus head apex in order to fit in the testing machine. An EMIC DL-3000 universal testing device (EMIC, Brazil) with a 5 kN load cell calibrated and measured by Instron Brazil Scientific Equipment (Certificate #17041003DF 10/04/2017) was utilized. The models were positioned lengthwise and axial load was applied at a deformation speed of 5.0 mm/min for displacement rate measurement (Fig 1). For axial load, forces were oriented and distributed vertically in the coronal and sagittal planes onto the proximal humerus head, similar to the investigation of He et al. [11].

In the control group, failure was considered to be varus collapse of the proximal fragment. Failure in the experimental groups was considered distal migration of the proximal fragment, with closure of the medial gap. The results were recorded in TESC 3.04 software (EMIC, Brazil), which generated real-time curves for applied force versus vertical displacement.

The stiffness of the bone models was calculated from the slope of the linear regions of the force/displacement curves, disregarding the region below 100 N, which was influenced by how the bone model was situated on the machine supports.

The data were statistically analyzed in a descriptive manner and presented in tables with the observations expressed as median, interquartile interval (Q1–Q3), minimum, maximum, graphic illustrations, and inferential statistics consisting of analysis of variance (ANOVA) and the Kruskal-Wallis test for multiple Dunn comparisons to verify whether there were significant differences in stiffness (N/mm) between the groups. The stiffness data for the bone models did not demonstrate normal Gaussian distribution after rejection of the normality hypothesis using the Shapiro-Wilk test (*p* = 0.05) and asymmetry in the graphic analysis of the histogram in the total sample. Significance was set at 5%, and the statistical analysis was conducted using SPSS version 20.0 software.

## Results

**Fig 2:**
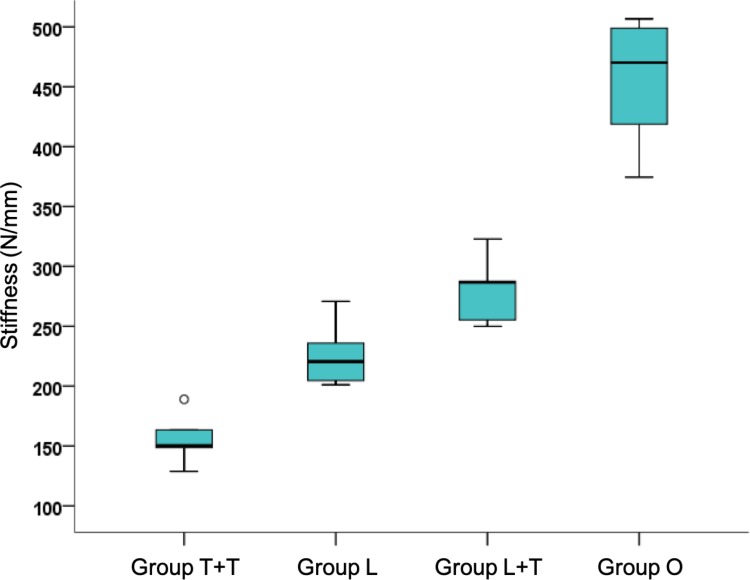
Stiffness (N/mm). Significantly lower stiffness values were seen in the T+T group than in groups L, L+T, and O (*p* = 0.01 for all). Group L demonstrated significantly lower stiffness than groups L+T (*p* = 0.03) and O (*p* = 0.01), and group L+T had significantly lower stiffness than group O (*p* = 0.01); the stiffness values can be represented as T+T < L < L+T < O. Group T+T: two non-locking one-third tubular plates positioned at right angles; Group L: locking anatomic plate positioned on the lateral surface of the humerus; Group L+T: two perpendicular plates, one locking and one one-third tubular; Group O: control.

A significant difference in stiffness (N/mm) between the study groups was seen according to the Kruskal-Wallis ANOVA (*p* = 0.001), as illustrated in Fig 2.

Table 2 provides the descriptive analysis of the stiffness values (N/mm) in each sample of the four groups (T+T, L, L+T, O), and the values behind the mean, standard deviation (SD), 1^st^ quartile (Q1), maximum, median, minimum, and 3^rd^ quartile (Q3) used to build Fig 2.

**Table 2:** Stiffness values (N/mm) in each sample of the four groups (T+T, L, L+T, O), and the values behind the mean, standard deviation (SD), 1^st^ quartile (Q1), maximum, median, minimum, and 3^rd^ quartile (Q3).

Sample	Group T+T *	Group L *	Group L+T *	Group O *
1	128,86	201,07	322,8	374,49
2	148,66	235,93	255,3	506,76
3	188,97	204,56	286,62	498,9
4	163,47	220,48	287,52	418,86
5	150,41	270,83	249,9	470,07
Mean	156,07	226,57	280,43	453,82
Standard deviation (SD)	22,16	28,37	29,36	56,16
1^st^ quartile (Q1)	148,66	204,56	255,30	418,86
Maximum	188,97	270,83	322,80	506,76
Median	150,41	220,48	286,62	470,07
Minimum	128,86	201,07	249,90	374,49
3^st^ quartile (Q3)	163,47	235,93	287,52	498,90

* All values are expressed in N/mm.

Table 3 provides the descriptive analysis of the stiffness values (N/mm) in the four groups (T+T, L, L+T, O), and the corresponding descriptive level (p value) for the statistical tests.

**Table 3:** Stiffness values (N/mm) in the four groups (T+T, L, L+T, O), and the corresponding descriptive level (p value) for the statistical tests.

Group	Median	IQI			minimum	maximum	p value ^*a*^	Significant differences ^*b*^
T+T	150	139	-	176	129	189	**0.001**	T+T ≠ L, L+T, and O
L	220	203	-	253	201	271		L ≠ L+T, and O
L+T	287	253	-	305	250	323		L+T ≠ O
O	470	397	-	503	374	507		

IQI: interquartile interval (Q1–Q3).

^a^ Kruskal-Wallis ANOVA.

^b^ Dunn’s multiple comparison test, at 5%.

Group T+T: two non-locking one-third tubular plates positioned at right angles; Group L: locking anatomic plate positioned on the lateral surface of the humerus; Group L+T: two perpendicular plates, one locking and one one-third tubular; Group O: control.

**Fig 3:**
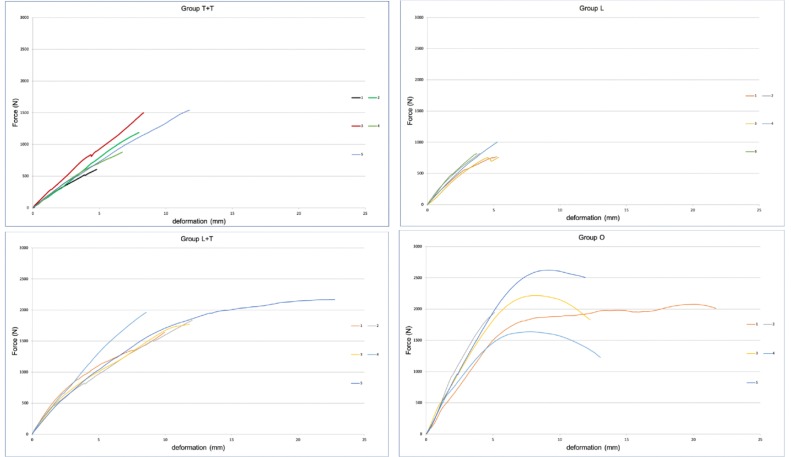
Stiffness (N/mm) of the bone models per group calculated from the slope of the linear regions of the force/displacement curves. A. Group T+T: two non-locking one-third tubular plates positioned at right angles; B. Group L: locking anatomic plate positioned on the lateral surface of the humerus; C. Group L+T: two perpendicular plates, one locking and one one-third tubular; D. Group O: control.

Dunn’s multiple comparison test found significantly lower stiffness values for group T+T than groups L, L+T, and O (*p* = 0.01 for all), and stiffness values for group L were significantly lower than for groups L+T and O (*p* = 0.03 and *p* = 0.01, respectively). Stiffness values were significantly lower for group L+T than group O (*p* = 0.01). Fig 3 provides the stiffness of the bone models per group calculated from the slope of the linear regions of the force/displacement curves.

**Fig 4:**
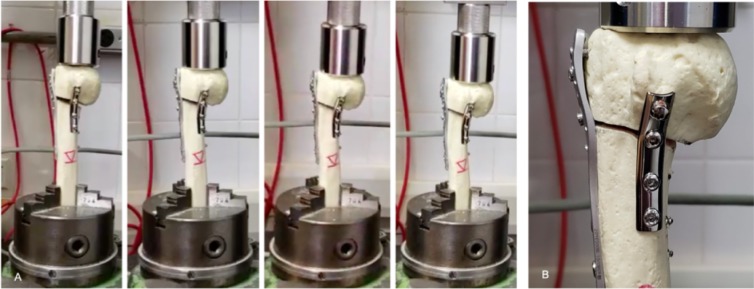
Sequence of mechanical testing in one model of Group L+T. A. Four phases identified during the test. Note that gradually humerus head suffered substantial varus collapse and both medial and lateral gap disappear; B. Mode of failure of Group L+T: there were obvious loosening of the proximal part of the lateral locking plate from the humerus head due to varus displacement. In addition, the two most proximal screws from the anteromedial nonlocking plate pushed out from the humerus head as well. There were no signs of breakage of any screws or plates nor hardware cut-out or cut-through.

Group T+T presented an unexpected behavior based on the previous study by Wanner et al. [10]. The unstable environment created by a 10-mm medial defect and a 5-mm lateral defect concentrated to much stress on the non-locked screws, producing varus collapse of the humerus head and screws pull-out, especially on the calcar screw. Both groups L and L+T showed no screw pull-out, however stiffness was significantly lower for group L. In L+T group, the stresses were dispersed largely from the lateral locking plate by adding a medial plate (Fig 4).

## Discussion

In humeral neck fractures without medial support, the combination of one locking anatomic lateral plate with a non-locking anteromedial plate produced the highest stiffness values in comparison with other configurations. In the current investigation, we were able to show both (1) addition of a non-locking anteromedial plate significantly increases construct’s axial stiffness if a lateral locking plate is used and (2) a locking lateral plate on its own improves construct’s axial stiffness significantly more than the combination of non-locking lateral plate and a non-locking anteromedial plate.

The addition of a non-locking anteromedial plate significantly increased construct’s axial stiffness in group L+T. He et al. observed similar performance, demonstrating that the stress on the lateral plate is dispersed by the presence of the medial plate [7,11]. But unlike this present study, those authors utilized a locking plate in the medial column, which increases costs related to the surgical procedure. In this experiment, a non-locking one-third tubular plate was used. The advantages of this hardware include affordability, low profile, better adaptation to the surface, and the ability to place screws at various angles in order to avoid contact with the screws from the lateral locking plate, which have a fixed angle. Theopold et al. also demonstrated that the use of a second plate alters load distribution [5]. In their experiment, they used an inverted non-locked one-third tubular plate in the bicipital groove. In clinical scenario, in order to use this construction, surgeon needs to cut the tendon of the long head of the biceps muscle, which can be seen as a potential limitation. Anyway, direct anteromedial plating support provided more effective dual-column stabilization only when a locked lateral plate was applied, a common finding in the studies by He et al., Theopold et al., and ours.

The use of two perpendicular non-locking plates, one lateral and one anteromedial as proposed by Wanner et al., results in lower stiffness against the deforming axial load, and is not sufficient to maintain reduction and avoid varus collapse when there is failure of medial support in proximal humeral fractures [10]. In a systematic review of the literature, Jabran et al. concluded that locking plate systems have greater pullout resistance and stiffness than non-locking plates in proximal humeral fractures [12]. When the groups which included locking plates on the lateral face (groups L and L+T) were compared, addition of the medial plate produced a clear improvement in mechanical stability. Greater loads were required in the L+T arrangement to provoke migration of the proximal fragment and collapse of the medial defect.

Some studies have indicated the importance of mechanical support in the medial region to maintain reduction in unstable proximal humeral fractures when osteosynthesis is performed with a locking lateral plate [5–9,13,14]. One of the most common strategies to increase stiffness of the anatomic lateral plate, particularly when a medial gap is present, is the use of a locking medial support screw or calcar screw. Gardner et al. and Zhang et al. showed that this hardware permits reduction to be maintained even in fractures where there is no medial cortical contact by decreasing interfragmental mobility and acting as a support against posterior-medial instability [3,6,9,14]. In this present study, a medial support screw was used in two of the arrangements with a locking lateral plate (groups L and L+T), but this was not seen as a primary cause of greater stiffness against deforming axial force. Even with the placement of this screw, a significant difference was seen between these groups (L < L+T), showing that the medial support screw as an isolated factor was not sufficient to reduce varus collapse at the head of the humerus. Recently, Zeng et al. concluded that the number of medial support screws is important when a lateral locking plate is used to treat proximal humerus fractures with lack of medial support [15]. They suggested that using two or more medial support screws in these situations decreases the risk that reduction will be lost and tends to provide better functional recovery of the shoulder. Despite the number of medial support screws used, medial support is an important factor in preventing post-operative complications [4–5,7,16].

In theory, placing the plate in the anteromedial region of the humeral head increases the risk of vascular injury, specifically to the ascendant and arched branches of the anterior humeral circumflex artery (AHCA) that penetrate the proximal region of the humerus and are important sources of blood supply to the humeral head. Knowledge about the location of these structures is crucial to ensure that the surgeon does not damage the vessels while positioning the implant [1]. Chen et al. observed that the average distances between the origin of the AHCA and the infraglenoid tubercle, the coracoid process, the acromion, and the mid-clavicular line were 26.9 mm, 49.2 mm, 67.0 mm, and 74.9 mm, respectively [17]. Anatomically, the medial, posterior-medial, and lateral regions are critical for the entry of the humeral circumflex artery branches, and should be avoided in placing retractors as well as implants [18]. Because the plate is positioned on the anteromedial face of the humeral head, there is less risk of intraoperative vascular damage. Additionally, Lambert showed that the head of the humerus can be completely revascularized, even when there is significant deviation or fragmentation of the posteromedial cortex, since significant anastomoses are distally present between the deltoid and ACHA [18]. As a result, at least four holes should be used for the medial plate, which should be located at the level of the insertion of the upper margin of the teres major muscle, just above the metaphyseal arterial ring. In our clinical experience, we advocate the use of a dual-plating technique, using a lateral locked plate and an anteromedial non-locked one-third tubular plate when a medial gap is present.

## Limitations

The main limitations of the current study are the use of plastic bone models, not conducting mechanical tests to replicate all the forces that act on proximal humerus, and not continuing the mechanical evaluation until catastrophic failure. First, plastic bone models were used because of the higher reproducibility they offer for testing experimental mechanics; they allow small differences to be described in detail, even in a small sample [19]. Stiffness and hardness are the two main structural properties which have been reported for artificial as well as cadaver humeri [19]. Studies using composite plastic models have demonstrated greater homogeneity, with lower standard deviation for stiffness and values within the limits of cadaver studies [20,21]. The same was seen in this present study, when the five intact specimens (group O) were tested identically to the other experimental groups and demonstrated homogeneous mechanical behavior among the specimens in terms of stiffness. In contrast, there is a lack of consensus regarding the most rigid anatomical plane in cadaver humeri, as significant heterogeneity has been observed between paired right and left humeri, as well as large standard deviations in values for stiffness and hardness [22–24]. The composites used in our experiment (3012BESP, Nacional Ossos, Brazil) were made in rigid polyurethane, with cortical and cancellous layers, and medullary canal of 7.0 mm in diameter. Although there is no specific study in the literature that have validated this humerus bone model, authors have been using other synthetic models from the same company, demonstrating homogeneous outcomes in biomechanical experiments for proximal femur fractures [25,26]. In addition, although we did not carry out a specific validation study of the model used, the five specimens in group O demonstrated homogeneous mechanical behavior and significantly higher stiffness than experimental groups, which seems to show its potential reproducibility for the type of investigation performed.

Secondly, reproducing axial deforming force is the simplest and most common mechanical test performed in studies assessing types of implants and fixation in models of proximal humeral fracture [12]. Traditionally in this type of experimentation, relative movements between the proximal and distal fragments are evaluated using a force/displacement curve, and this technique was utilized in the present study. The existence of a medial gap zone promotes the emergence of a vector associated with deformation, combining axial force and the bending moment, and its failure criterion is closure of the medial cortical defect [27]. Other authors have used the same parameter to experimentally simulate the existence of medial comminution [27–29].

Thirdly, none of the hardware assemblies in our study was tested until catastrophic failure of the fixation was reached. In biomechanical scenario, load to failure is related both to material’s ultimate tensile strength and ultimate compressive strength. In clinical scenario, catastrophic failure is important to better understand the mechanical performance of an osteosynthesis, thus reducing the risk of construct failure in daily activities, such as rising out of a chair or crutching weight-bear in the early postoperative period [11]. In the current investigation, we could not damage the implants because they were all provided for experimentation, on the condition that they would be returned undamaged. It should be noted, however, that the addition of the medial plate was the only differentiating factor between the three assemblies we investigated, and was directly associated with increased stiffness. As mentioned previously, the stress on the lateral plate is dispersed by the presence of the medial plate, and it can consequently be inferred that its presence potentially increases the lateral plate's resistance to catastrophic failure [7,11]. This factor may be affected by different types of non-locking and locking medial plates. Thus, future studies could investigate this biomechanical aspect and also assess the role of a locking one-third tubular plate in an anteromedial position.

## Conclusion

The combination of a locking lateral plate and a non-locking anteromedial plate is the construction which provides the greatest stiffness values for fixation of a fracture at the proximal end of the humerus in two parts with a medial gap. Mechanical benefits of medial support with a second non-locking antero-medial plate seems to be related with better construct stability in terms of strength and fatigue, potentially reducing the risk of varus collapse of the humerus head and fracture healing disturbances.
